# Improved Retinal Microcirculation in Mild Diabetic Retinopathy Patients Carrying MTHFR Polymorphisms Who Received the Medical Food, Ocufolin^®^

**DOI:** 10.2147/OPTH.S358753

**Published:** 2022-05-16

**Authors:** Zhiping Liu, Hong Jiang, Justin H Townsend, Jianhua Wang

**Affiliations:** 1 Ophthalmic Center, The Second Affiliated Hospital of Guangzhou Medical University, Guangzhou, Guangdong, People’s Republic of China; 2 Department of Ophthalmology, Bascom Palmer Eye Institute, University of Miami Miller School of Medicine, Miami, FL, USA; 3 Department of Neurology, University of Miami Miller School of Medicine, Miami, FL, USA

**Keywords:** diabetic retinopathy, homocysteine, l-methylfolate, medical food, MTHFR, Ocufolin^®^, retinal tissue perfusion, vessel density

## Abstract

**Purpose:**

To evaluate the effects of Ocufolin^®^ on retinal microcirculation in patients with mild diabetic retinopathy carrying MTHFR polymorphisms.

**Methods:**

In a prospective, case-controlled study, eight patients with mild diabetic retinopathy and MTHFR polymorphisms and 15 normal controls (NC) were recruited. MTHFR polymorphisms were subtyped as normal, C677T, or A1298C. Best-corrected visual acuity (BCVA) was evaluated. Retinal blood flow velocity (BFV) was measured using Retinal Function Imager. Retinal tissue perfusion (RTP, blood flow rate per inner retinal volume) was calculated within a 2.5 mm diameter circle centered on the fovea. The eight retinopathy patients received Ocufolin^®^ for 6 months, and their imaging was performed at baseline, 4 months, and 6 months. The NC group was imaged once.

**Results:**

BCVA and vascular indices of DR + PM patients at baseline were below those of NC and improved after Ocufolin^®^ administration. Compared to baseline, DR + PM patients had significantly improved BCVA during the follow-up period (P < 0.05). RTP and arteriolar BFV were significantly increased at 6 months (P < 0.05), approaching NC.

**Conclusion:**

Ocufolin^®^ may be effective in improving both visual acuity and retinal microcirculation in patients with DR + PM. Further studies with increasing sample size, and longer duration, including cases with severe DR, are needed.

## Introduction

Diabetic Mellitus (DM) is a major public health problem worldwide due to its complications. Diabetic retinopathy (DR), a microvascular complication of DM, is the leading cause of blindness in working-age adults.^1^ In DR eyes, the loss of pericytes results in the formation of microaneurysms.^2^,^3^ Retinal circulation is further affected by the proliferation of vascular endothelial cells with subsequent thickening of the vessel basement membrane.^4^,^5^ Studies of DR have shown that capillary network dropout and resultant retinal ischemia progress over time.^6^,^7^ Additionally, our previous study found that retinal tissue perfusion (RTP), vessel density, and the thickness of the retinal fiber layer in patients with mild DR were significantly reduced.^8^ In that study, RTP had the highest discrimination power to detect early-stage DR. Microcirculation impairment (ie, RTP) in mild DR provides a promising biomarker to evaluate the treatment effect of medical therapy.^8^

DM is a chronic disease associated with a number of micro-and microvascular complications that increase the morbidity and mortality of patients. The risk of diabetic complications has a strong genetic component. Studies have shown that single-nucleotide polymorphisms (SNPs) in miRNA genes have been associated with diabetes and atherosclerotic cardiovascular disease in different populations.^9^ Additionally, significant associations of variants in the CAT, FTO, and UCP1 genes with DR and nephropathy are known.^10^

Hyperglycemia induces proliferative alterations in DR typically accompanied by other risk factors for vascular disorders. Recently, the role of hyper-homocysteinemia (Hhcy) in the development of DR has been reported.^11^ Homocysteine (Hcy) is a sulfur-containing intermediate metabolite between cysteine and methionine. Excess Hcy is vasotoxic. Excess plasma Hcy is frequently reported in MTHFR reduced function mutations carriers.^12^ MTHFR is an enzyme involved in the remethylation of folate, a cofactor for the conversion of homocysteine to methionine. Two common reduced function MTHFR polymorphisms have been described: a mutation coded at nucleotide 677 (C677T) and another at nucleotide 1298 (A1298C). These polymorphisms are associated with reduced enzyme activity, resulting in decreased methylation of Hcy and its accumulation in plasma and tissues.^13^

Previous studies found that elevated serum Hcy is associated with retinal artery constriction, reduced retinal blood flow, endothelial dysfunction, ischemia, and retinal ganglion cell death.^14–16^ Clinical studies have demonstrated that MTHFR folate polymorphisms can contribute to the progression of DR, especially in patients with poorly controlled blood glucose.^13^,^17^ Maeda et al proposed that personalized medicine for diabetes mellitus, based on a better understanding of MTHFR gene polymorphisms and their ramifications, has the potential to improve understanding and outcomes for diabetic retinopathy.^17^ However, there are few studies of retinal microcirculation after medical therapy based on Hcy or MTHFR polymorphism status.

We have recently reported improvements in photographically documented retinopathy observed in a case series of patients with DR + PM following the administration of Ocufolin^®^ (Global Healthcare Focus, Montgomery, AL, USA, a medical food).^18^ The ingredients of Ocufolin^®^ have been described in our previous studies.^19^,^20^ We have demonstrated improved conjunctival microcirculation after the administration of Ocufolin^®^. Additionally, we have also shown that a six-month intake of Ocufolin^®^ can reverse microvascular structural damage and improve visual acuity in patients with DR+PM.

Based on theoretical mechanisms, case series reports, and a previous study, we proposed that Ocufolin^®^ would have beneficial effects on retinal microcirculation in mild DR patients with DR + PM. This might suggest a new approach for addressing ischemia in patients with DR + PM.

## Methods

### Collection of Study Subjects

This study complies with the ethical principles of the Declaration of Helsinki. Ethical clearance for the study was obtained from the Research Subjects Review Board office of the University of Miami (ID: 20070492, approved on Jan 18, 2017). Informed consent was obtained from all participants before enrollment. Participant eligibility was determined at a screening appointment according to the inclusion and exclusion criteria outlined in the study protocol. The inclusion criteria were: mild DR without other retinal vascular diseases; clear corneas and crystalline lens; initial visual acuity of 20/80 or better; and presence of the MTHFR reduced function polymorphisms: A1298C and, or, C677T. The exclusion criteria were: glaucoma, visually significant cataract, other retinal diseases, infectious diseases, cardiovascular disease, dementia, cerebrovascular diseases, cancer, and multiple sclerosis. The patients underwent a complete baseline fundus examination. Mild DR was diagnosed by a retinal specialist (JT). The diabetes diagnosis was made based on American Diabetes Association (ADA) criteria and retinopathy diagnosis according to the American Academy of Ophthalmology Retina/ Vitreous Panel.^21^,^22^ Between August 2017 and January 2020, forty-two subjects were recruited for this prospective study at the Bascom Palmer Eye Institute, University of Miami Miller School of Medicine. Twenty-seven patients with mild DR were screened for MTHFR polymorphisms. Genetic testing (MTHFR polymorphisms) was performed by MyGenetx Laboratory, LLC (Franklin, TN). Other blood tests, including homocysteine and HbA1c, were performed by Quest Diagnostics (Secaucus, NJ). The refractive errors of the subjects ranged from −6 to +3 diopters. Thirteen DR + PM patients were eligible. One patient declined to participate, while four patients failed to complete all follow-up visits. Eight patients completed all visits. Fifteen gender and age-matched healthy individuals were recruited as normal controls (NC).

Each patient with DR + PM took Ocufolin^®^ for a total of six months (6M) according to the following dose regimen: one capsule orally each morning for the first week, then two capsules each morning for the second week, and then three capsules for the rest of the six months. Ocufolin^®^ intake was confirmed when patients came back at 4M (second visit) and 6M (final visit). Best-corrected visual acuity (BCVA, LogMAR), slit-lamp photography, retinal microcirculation markers, systolic blood pressure (SBP), diastolic blood pressure (DBP), mean artery pressure (MAP), heart rate (HR), and mini-mental status (MMSE) were evaluated at these time points. The data of NC were collected once.

### Measurement of Retinal Microcirculation

As described in previous studies,^8^,^23^ retinal arteriolar blood flow velocity (ABFV) and venular blood flow velocity (VBFV) were measured using the Retinal Function Imager (RFI, model 3000, Optical Imaging, Rehovot, Israel). Images were acquired after the pupil was dilated with tropicamide 1%. Image acquisition was synchronized with the heartbeat. BFV was measured based on the motion of erythrocytes in a sequence of eight consecutive fundus photos recorded at 60 frames per second.

**Figure 1:**
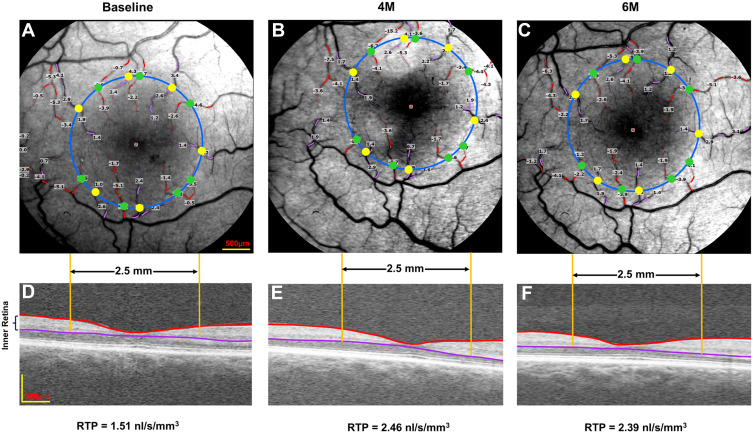
Representative images of retinal blood flow and tissue volume in a patient. A retinal function imager was utilized to acquire retinal blood flow at baseline (**A**), 4M (**B**), and 6M (**C**). The arterioles and venules were marked in red with negative values and pink with positive values, respectively. The total macular flow was the sum of all flow in the arterioles (green dots) and venules (yellow dots), which crossed the 2.5 mm diameter circle centered on the fovea. The volumes of the inner retina at baseline (**D**), 4M (**E**), and 6M (**F**) were acquired from a 6×6 mm optical coherence tomography angiography scan image. RTP was calculated as retinal blood flow rate per inner retinal volume within a 2.5 mm diameter circle centered on the fovea.

**Abbreviations**: 4M, 4 months; 6M, 6 months; RTP, retinal tissue perfusion.

Images with a field of view (FOV) of 4.3×4.3 mm (20 degrees) or 7.3×7.3 mm (35 degrees) were obtained. BFV was calculated from four or more serial images centered on the fovea. Based on a previous study,^24^ the conversion factors of ABFV and VBFV from the FOV of 35 degrees to the FOV of 20 degrees were 0.95 and 0.92, respectively. As described in previous studies,^8^,^23^ RTP was calculated as retinal blood flow per inner retinal volume within a 2.5 mm diameter circle centered on the fovea. The inner retinal volume within a 2.5 mm diameter circle centered on the fovea was calculated from a 6×6 mm volumetric scan of optical coherence tomography (OCTA, Optovue, Fremont, CA, USA) (Figure 1).

### Statistical Analysis

All values are presented as mean ± standard deviation. All analyses were performed using IBM SPSS Statistics software package (version 25, IBM Corp., Armonk, NY, USA). Descriptive statistics were used to summarize patient demographic and clinical information. The main outcome, RTP, was compared between visits. A generalized estimating equation (GEE) model was used to evaluate differences in microcirculation measurements. P values less than 0.05 were considered statistically significant.

## Results

### Study Population and Baseline Clinical Characteristics

Table 1 reports the baseline characteristics of the eight DR + PM patients and fifteen NC subjects. The majority of patients were male (75.0%) with well-controlled hypertension (62.5%). The mean HbA1c in DR + PM patients was 7.6 ± 0.9% (range, 6.9–8.1). The mean diabetic duration was 14.5 ± 7.3 years (range, 5–25). Four patients carried the C677T mutation, two carried the A1298C mutation, and two carried both C677T/A1298C (compound heterozygous mutation). The healthy control individuals did not undergo blood chemistry or genetic testing.

**Table 1:** Demographics of the Patients with DR + PM at Baseline and Normal Subjects

	DR + PM	NC	P-value
Subjects	8	15	
Race/ethnicity	White 6, Black 1, Asian 1	White 6, Black 1, Asian 8	
Eyes	16	15	
Sex (Male/Female)	6:2	5:10	0.057 (χ^2^ test)
Age (Years)	58.3±6.8	51.3±10.6	0.070 (*t*-test)
Duration of Diabetes (Years)	14.5±7.3	NA	NA
Hypertension (n, %)	5 (62.5%)	NA	NA
HbA1C (%)	6.9±0.8	NA	NA
Hcy (µmol/L)	12.2±4.2	NA	NA
MTHFR mutation	C677T/A1298C	NA	NA

**Abbreviations**: DR + PM, mild diabetic retinopathy patients with methylenetetrahydrofolate reductase polymorphisms; NC, normal controls; HbA1c, glycosylated hemoglobin; Hcy, homocysteine; MTHFR, methylenetetrahydrofolate reductase.

### Effect of Ocufolin^®^ on DR + PM Patients

During the entire follow-up period, there were no remarkable alterations of MMSE, MAP, DBP, and SBP in DR + PM patients after administration of Ocufolin^®^.

**Figure 2:**
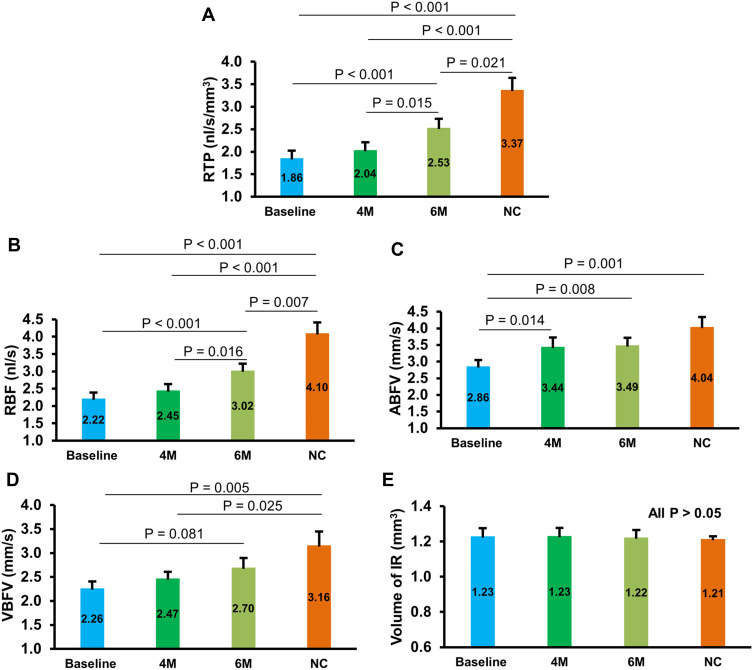
Changes of retinal microcirculation and corresponding tissue volume in DR + PM patients during follow-up period. RTP (**A**), RBF (**B**), and ABFV (**C**) of DR + PM patients at 6M were remarkably higher than those of baseline (all P < 0.05). RTP of DR + PM patients at 6M was significantly higher than those in 4M (P < 0.05, (**A**). Although no significant increase was observed in VBFV of DR + PM patients between baseline and 6M, a tendency toward the elevation of VBFV was seen at 6M (P = 0.081, (**D**). At baseline, RTP was lower in DR+PM patients, increased at 4M and 6M but not reached NC levels (all P < 0.001). However, no significant difference was observed in IR volume at any time points between DR + PM and NC groups (all P > 0.05, (**E**)). DR + PM, mild diabetic retinopathy patients with methylenetetrahydrofolate reductase polymorphisms.

**Abbreviations**: 4M, 4 months; 6M, 6 months; NC, normal controls; RTP, retinal tissue perfusion; RBF, retinal blood flow; ABFV, arteriolar blood flow velocity; VBFV, venular blood flow velocity; IR, inner retina.

As would be expected, the baseline RTP values of DR + PM patients were lower than normal controls. Following administration of Ocufolin^®^, significant improvements were found in retinal microcirculation over time in DR + PM patients (Figure 2). At 6M, RTP and ABFV were increased significantly over baseline (all P < 0.05). Additionally, RTP was remarkably higher at 6M than at 4M (P < 0.05). An increasing trend of VBFV in DR + PM patients (P = 0.081) was seen at 6M as compared to baseline. At 4M and 6M, RTP values increased in DR + PM patients, approaching but not reaching normal blood flow levels (all P < 0.001, Figure 2).

**Figure 3:**
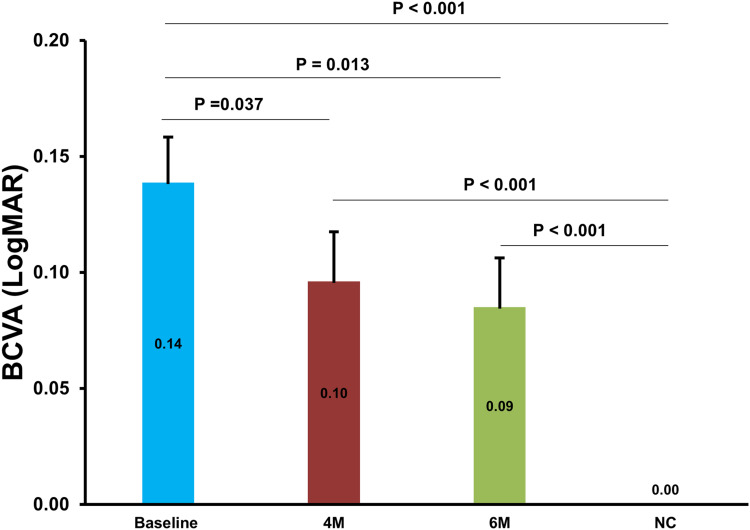
Changes of BCVA (LogMAR) in DR + PM patients at baseline, 4M, and 6M. The effect of Ocufolin^®^ on BCVA (LogMAR) was significant. Compared to baseline, BCVA of DR + PM patients at 4M and 6M significantly and progressively improved, although it did not reach that of the NC. DR + PM, mild diabetic retinopathy patients with methylenetetrahydrofolate reductase polymorphisms.

**Abbreviations**: BCVA, best-corrected visual acuity; NC, normal controls; 4M, 4 months; 6M, 6 months.

Similarly, compared with baseline, the DR + PM group showed significant improvement in BCVA at 4M and 6M. At 6M, BCVA in the DR + PM group approached but did not reach the BCVA of NC (all P < 0.05; Figure 3).

## Discussion

To the best of our knowledge, this is the first study to show significant improvement in visual acuity and retinal microcirculation in any cohort of diabetic retinopathy patients following the administration of Ocufolin^®^. The current study revealed increased microcirculation and microvasculature together with improved BCVA at 4M and 6M after Ocufolin^®^ intake in these patients carrying the common MTHFR polymorphisms.

MTHFR mutations and marginal nutrition affect vitamin and mineral homeostasis in diabetic retinopathy patients.^25^ Raising the levels of antioxidants B6, B12, and L-methylfolate in the central nervous system (CNS) is known to reduce mitochondrial oxidative stress while lowering homocysteine.^18^,^26^ It is likely that a similar mechanism is at work in these DR + PM patients with impaired homocysteine (Hcy) metabolism. We interpret these findings to show that Ocufolin^®^ improves visual function by increasing blood flow and reducing ischemia. These benefits might be extended with a longer duration of supplementation.

Our previous case series reports documented anatomic improvement of visible retinopathy in DR + PM patients after intake of Ocufolin^®^. Our findings are evidence that Ocufolin^®^ associated improvements in retinal microcirculation may be the mediators of vision improvement.^18^ In DR + PM patients, such as those tested in the present study, medical supplements like Ocufolin^®^ may provide important nutritional entities (ie, lutein, zeaxanthin carotenoids, vitamin C, vitamin D, natural vitamin E complex, zinc, copper, n-acetyl cysteine, and complexes of B1, B2, B6, L-methylfolate, and methyl B12) sufficient to improve blood flow and visual acuity while making it simple for the patient to obtain all of these benefits.

Ocufolin^®^ contains several ingredients that have been shown to have beneficial effects on the ischemic retina. Thiamine (vitamin B1) protects vascular endothelial and retinal cells from harmful advanced glycation endproducts.^27^ The vitamins B2, B6, folate, and B12 are important co-factors for converting Hcy into methionine, improving insulin resistance, lowering blood pressure, and enhancing DNA methylation.^28^ Removing folic acid and boosting L-methylfolate intake increases CNS and retinal active L-methylfolate, lowers Hcy, and decreases oxidative stress, thus reversing the downstream metabolic effects of the MTHFR polymorphisms.^26^ Vitamin D enhances L-methylfolate uptake. Higher serum concentrations of Vitamin D are associated with less risk of DR.^29^ The AREDS formulation of antioxidants and zinc reduces diabetic retinopathy progression in animal models.^30^ Glutathione is the main mitochondrial antioxidant, improving glucose metabolism by reducing oxidative stress. Vitamin C, E, selenium, and n-acetyl cysteine increase glutathione by recycling or by increasing its synthesis.^31^,^32^

There are several limitations to our present study. First, the sample size is relatively small. However, this was the first study to demonstrate and quantify the improvement of impaired retinal tissue perfusion in patients with mild DR carrying MTHFR polymorphisms following administration of antioxidants and L-methylfolate. Although there were only eight patients included, we found progressive improvement in RTP from baseline to 6M, suggesting that these numbers were sufficient to address the study question. Second, it is likely that a longer trial would show further improvements in visual acuity and retinal microcirculation. A previously published retrospective series, including moderate to severe DR patients, appeared to show anatomic improvement with longer-term follow-up.^18^ Third, we did not examine the level of Hcy at 4M and 6M, which may be an essential biomarker to indicate the alterations of the retinal hemodynamics. Of note, Schmidl et al reported a 30% reduction in serum homocysteine in a cohort of diabetics after a 3-month intake of a single Ocufolin^®^.^33^ Fourth, we did not perform blood biochemistry, hematology, or genetic testing on the healthy individuals who formed the normal controls, some of whom likely carried the MTHFR polymorphisms. Finally, we did not include diabetes without diabetic retinopathy, severe non-proliferative diabetic retinopathy, and proliferative diabetic retinopathy in the present study. The present study focused on the changes of early mild DR. Further studies should measure Hcy at the end of the trial and include prediabetes, diabetes without DR, and moderate to severe stages of DR, which may further enrich our knowledge on retinal tissue perfusion in DR and provide clinicians with new tools to address DR.

In summary, Ocufolin^®^ improved retinal microcirculation in patients with diabetic retinopathy and MTHFR polymorphisms. Further studies with larger sample sizes, longer duration, more parameters, and later stages of diabetic retinopathy may further validate the efficacy of Ocufolin^®^ as an agent to reverse diabetic retinopathy with associated vision and perfusion loss.
